# Effects of increased N and P availability on biomass allocation and root carbohydrate reserves differ between N‐fixing and non‐N‐fixing savanna tree seedlings

**DOI:** 10.1002/ece3.4289

**Published:** 2018-07-30

**Authors:** Varun Varma, Arockia M. Catherin, Mahesh Sankaran

**Affiliations:** ^1^ Ecology and Evolution Group National Centre for Biological Sciences (NCBS) Tata Institute of Fundamental Research (TIFR) Bangalore India; ^2^ Department of Biosciences University of Exeter Exeter UK; ^3^ School of Biology University of Leeds Leeds UK

**Keywords:** nodulation, nutrient deposition, plant functional groups, root carbohydrate, root‐shoot ratio, savannas, tree recruitment, tropical dry forests

## Abstract

In mixed tree‐grass ecosystems, tree recruitment is limited by demographic bottlenecks to seedling establishment arising from inter‐ and intra‐life‐form competition, and disturbances such as fire. Enhanced nutrient availability resulting from anthropogenic nitrogen (N) and phosphorus (P) deposition can alter the nature of these bottlenecks by changing seedling growth and biomass allocation patterns, and lead to longer‐term shifts in tree community composition if different plant functional groups respond differently to increased nutrient availability. However, the extent to which tree functional types characteristic of savannas differ in their responses to increased N and P availability remains unclear. We quantified differences in above‐ and belowground biomass, and root carbohydrate contents in seedlings of multiple N‐fixing and non‐N‐fixing tree species characteristic of Indian savanna and dry forest ecosystems in response to experimental N and P additions. These parameters are known to influence the ability of plants to compete, as well as survive and recover from fires. N‐fixers in our study were co‐limited by N and P availability, while non‐N‐fixers were N limited. Although both functional groups increased biomass production following fertilization, non‐N‐fixers were more responsive and showed greater relative increases in biomass with fertilization than N‐fixers. N‐fixers had greater baseline investment in belowground resources and root carbohydrate stocks, and while fertilization reduced root:shoot ratios in both functional groups, root carbohydrate content only reduced with fertilization in non‐N‐fixers. Our results indicate that, even within a given system, plants belonging to different functional groups can be limited by, and respond differentially to, different nutrients, suggesting that long‐term consequences of nutrient deposition are likely to vary across savannas contingent on the relative amounts of N and P being deposited in sites.

## 1 INTRODUCTION

The structure and functioning of mixed tree‐grass ecosystems, such as savannas, are governed by both bottom‐up (e.g., water and nutrient availability) and top‐down drivers (e.g., fire and herbivory) (Ekblom & Gillson, 2010; February, Higgins, Newton, & West, 2007; Frost et al., 1986; Higgins, Bond, & Trollope, 2000; Sankaran, Ratnam, & Hanan, 2004, 2008; Scholes & Archer, 1997). Among bottom‐up drivers, the importance of water availability in regulating savanna structure and dynamics is well recognized (Lehmann et al., 2014; Sankaran et al., 2005). However, the influence of nutrient availability on the functioning of these ecosystems is less clear (February & Higgins, 2010; Sankaran et al., 2008; van der Waal et al., 2011). Given that savannas and dry forest ecosystems are anticipated to be particularly vulnerable to future global change drivers including nutrient deposition (Sala et al., 2000), understanding the impacts of enhanced nutrient availability on vegetation dynamics of mixed tree‐grass systems is important, both to assess their future trajectories and to develop appropriate management strategies.

Atmospheric nutrient deposition, a major global change driver, has dramatically increased the quantities of plant available nitrogen (N) and phosphorus (P) cycling through ecosystems across the globe (Bennett, Carpenter, & Caraco, 2001; Galloway et al., 2004, 2008; Phoenix et al., 2006; Vitousek, 1994; Vitousek et al., 1997). This increased availability of N and P has the potential to impact both the structure and composition of mixed tree‐grass communities. For example, in African savannas, tree basal area has been shown to be negatively correlated with soil N across broad scales (Sankaran et al., 2008), suggesting that enhanced N availability can potentially alter vegetation structure by shifting communities towards more grassy states. Nutrient deposition can also affect the composition of tree communities if increased nutrient availability has differing effects on the dominant tree functional types that characterize these ecosystems, namely, N‐fixers and non‐N‐fixers.

N‐fixing and non‐N‐fixing species differ inherently in their nutritional requirements, leaf chemistry, and physiology (Pearson & Vitousek, 2002; Powers & Tiffin, 2010; Vitousek, Menge, Reed, & Cleveland, 2013; Vitousek et al., 2002), and thus may be expected to respond differently to increases in N and P availability (Barbosa et al., 2014; Cramer, Chimphango, Van Cauter, Waldram, & Bond, 2007; Cramer, Van Cauter, & Bond, 2010; Khurana & Singh, 2004). N‐fixing plants have tissues that are richer in N than non‐N‐fixers due to their association with N‐fixing bacteria in root nodules. Additionally, plants that invest in symbiotic N_2_ fixation also have greater requirements for P, as a consequence of the high ATP requirements for nodule development and function, and the high P content of nodule bacteroid membranes (Graham & Vance, 2003; Vance et al. 2000; Vitousek et al., 2002). N‐fixing plants are thus characterized by an N and P demanding lifestyle compared to non‐N‐fixers (Pearson & Vitousek, 2002; Vitousek et al., 2002, 2013), and therefore, might be expected to show smaller biomass increases for a given amount of added N and P relative to non‐N‐fixers. Nodulation, although an advantage in N limited soils, incurs a substantial energetic cost on the plant (Gutschick, 1981; Sprent, 1999; Vitousek & Howarth, 1991; Vitousek, Porder, Houlton, & Chadwick, 2010; Vitousek et al., 2002), and studies have previously shown that N‐fixers reduce investment in root nodules with increasing N availability (Barron, Purves, & Hedin, 2010; Sanginga, Mulongoy, & Ayanaba, 1988). N‐fixing savanna species have also been shown to reduce investment in nodulation in the face of reduced competition from grasses, and as seedlings grow (Cramer et al., 2007, 2010). Reduced investment in nodulation under conditions of increased nutrient availability can free up additional resources that can be diverted towards growth in N‐fixers. However, whether or not such effects translate to greater biomass increases in N‐fixers relative to non‐N‐fixers that have a lower N and P demand is unclear.

Besides altering growth, increased nutrient availability can also influence how tree seedlings respond to fire, a major determinant of vegetation structure in mixed tree‐grass ecosystems. The ability of both tree seedlings and juveniles to survive recurring fires by resprouting is key to their persistence and eventual recruitment into the canopy as reproductively mature adults (Bond, 2008; Bond & van Wilgen, 1996; Hanan, Sea, Dangelmayr, & Govender, 2008; Higgins et al., 2000; Sankaran et al., 2004, 2005; Schutz, Bond, & Cramer, 2009). This capacity to resprout, and thus, persist within the fire trap is contingent on seedlings being able to allocate sufficient resources belowground and invest in root carbohydrate reserves, which are remobilized to support the cost of postfire resprouting (Bell, 2001; Bell & Ojeda, 1999; Bond, Midgley, Woodward, Hoffman, & Cowling, 2003; Clarke & Knox, 2009; Clarke et al., 2013; Hermans, Hammond, White, & Verbruggen, 2006; Hoffmann, Bazzaz, Chatterton, Harrison, & Jackson, 2000; Knox & Clarke, 2005; Lamont & Wiens, 2003; Pate, Froend, Bowen, Hansen, & Kuo, 1990; Ryle, Arnott, & Powell, 1981; Schwilk & Ackerly, 2005; Verdaguer & Ojeda, 2002; Vesk & Westoby, 2004; Wang et al., 2015; Wigley, Cramer, & Bond, 2009). Allocation of resources belowground has been shown to be influenced by nutrient availability (Hermans et al., 2006; Knox & Clarke, 2005; Tilman, 1988; Wang et al., 2015), and plants in general tend to reduce belowground investment, that is, lower root–shoot ratios (henceforth, R:S ratios), and decrease root carbohydrate reserves with increasing N and P availability (Clarke & Knox, 2009; Hermans et al., 2006; Ryle et al., 1981; Wang et al., 2015). Differences between species and functional groups in allocation to belowground resources and root carbohydrate reserves with increasing nutrient availability can significantly alter postfire survival and the composition of the regenerating community. Such functional group level differences have previously been quantified, for example, between evergreen and deciduous savanna tree species (Tomlinson et al., 2013), but few studies have thus far evaluated how N‐fixers and non‐N‐fixers differ in their allocation patterns with N and P addition. Given their greater N and P demand, we expected N‐fixers to show greater relative investment in roots, as well as have higher root carbohydrate storage contents, when compared to non‐N‐fixers.

In this study, we quantified the effects of N and P fertilization on biomass accumulation, biomass partitioning, and root storage carbohydrate content in seedlings of multiple tree species characteristic of savanna and tropical dry forests in peninsular India. We chose these responses as they play an important role in determining the competitive ability of tree seedlings, as well as their ability to survive fires, a key disturbance agent in this ecosystem. We examined how responses differed between N‐fixers and non‐N‐fixers, and additionally, quantified changes in nodulation in N‐fixers when fertilized. We expected: (a) enhanced nutrient availability to lead to increased biomass accumulation in both functional groups, but the magnitude of increase to be greater amongst non‐N‐fixers, (b) N‐fixers to have greater baseline R:S ratios, as well as root carbohydrate content compared to non‐N‐fixers, and (c) nutrient addition to reduce R:S ratios and root carbohydrate content in both functional groups, with greater reductions in non‐N‐fixers.

## 2 MATERIALS AND METHODS

The experiment was conducted at a field site in the village of Hosur, located in Mysore district of the southern Indian state of Karnataka. A total of 13 commonly occurring savanna and tropical dry forest tree species were selected based on published sources (Kodandapani, Cochrane, & Sukumar, 2008; Kumar & Shahabuddin, 2005; Puyravaud, Pascal, & Dufour, 1994; Sagar & Singh, 2004) and included six N‐fixers *(Acacia catechu* (L.f.) Willd.*, Acacia ferrugin*ea DC.*, Acacia leucophloea* (Roxb.) Willd.*, Albizia amara* (Roxb.) B.Biovin.*, Albizia lebbeck* (L.) Benth. an*d Dalbergia latifolia* (Roxb.)), and seven non‐N‐fixers (*Lagerstroemia indica* (L.)*, Lagerstroemia speciosa* (L.) Pers.*, Phyllanthus emblica* (L.)*, Sapindus emarginatus* Vahl.*, Terminalia arjuna* (Roxb. ex DC.)*, Terminalia bellirica* (Gaertn.) Roxb., and *Zizyphus jujuba* Mill.).

Three‐week old seedlings were procured from the Foundation for the Revitalisation of Local Health and Tradition (FRLHT), Bangalore, transported to the field site and allowed to acclimatize to local conditions for 1 week. After the acclimation period, seedlings were transplanted into 20 L nursery polybags containing a 1:1 mixture of sand and local soil (July 2013). Each polybag contained only one seedling and was large enough that seedlings did not become pot‐bound during the course of the experiment. Average total C and N content of the sand–soil mix was 3.94 g/kg and 0.51 g/kg, respectively (Leco TrueSpec CN analyzer), and average total P content was 0.13 g/kg (Thermo iCAP 6300 ICP—OES dual view spectrophotometer). Seedlings in polybags were arranged in a uniform grid at the field site, with a minimum spacing of 40 cm between neighboring stems. Species treatment combinations were randomized within this grid to avoid spatial clustering. Each individual was assigned to one of four nutrient treatments—Control (no nutrient addition), N+ (5 g N), P+ (0.5 g P), and NP+ (5 g N and 0.5 g P). N and P were added to polybags as solutions of urea and single superphosphate (SSP), respectively, in three separate applications two, four, and 6 weeks after transplant. Seedlings were watered regularly to prevent water stress. Final sample sizes for each species‐treatment combination ranged from five to 14 individuals (see Supporting information Table S1 for exact number of replicates), with a total of 604 individuals in the entire experiment. Unequal sample sizes were a consequence of species‐level differences in seedling availability and differential seedling mortality due to transport and transplant stress.

All individuals were harvested 6 months after transplant (January, 2014). Shoots and roots were separated, and for the N‐fixers, root nodules were collected. Samples were transported to the National Centre for Biological Sciences, Bangalore, where they were oven dried for 5 days at 60°C before being weighed. At the time of weighing root samples, a section of the primary root from a subset of individuals, ranging from two to six individuals per species‐treatment combination (see Supporting information Table S1b for number of replicates), was extracted to estimate percent storage carbohydrates, that is, nonstructural carbohydrates (NSC), using the phenol‐sulfuric acid assay (Buysse & Merckx, 1993; Wigley et al., 2009). Ground root samples were digested in 3% HCl, followed by the addition of 27% phenol and concentrated sulfuric acid to the supernatant of the acid digest. The intensity of the resulting colour reaction was measured using a spectrophotometer at 490 nm, and sample carbohydrate content was estimated against a standard curve of glucose.

Responses of seedling total‐, above‐, and belowground biomass, R:S ratios and root carbohydrate content to fertilization were analyzed using linear mixed effects models, implemented using the *lme4* package (Bates, Maechler, Bolker, & Walker, 2014) in R (R Core Team 2014). Predictor variables included nutrient treatment, plant functional group, and the interaction between the two, with species identity included as a random factor as the focus of the analyses were to find generalizations at the functional group level, while accounting for intrinsic differences between species in the measured parameters. Significance tests of the fixed effects were carried out using Satterthwaite's approximation for degrees of freedom implemented within the *lmerTest* package (Kuznetova, Brockhoff, & Christensen, 2014). The nodule mass data for N‐fixers included a large number of zeros for the nutrient addition treatments resulting in a skewed distribution which did not match criteria to be considered a zero inflated distribution. Hence, for this analysis, we used species‐treatment means of nodule mass as the response variable within a linear mixed effects model, where nutrient treatment was the only predictor, and species identity included as a random factor.

## 3 RESULTS

### 3.1 Biomass accumulation and allocation

**Figure 1:**
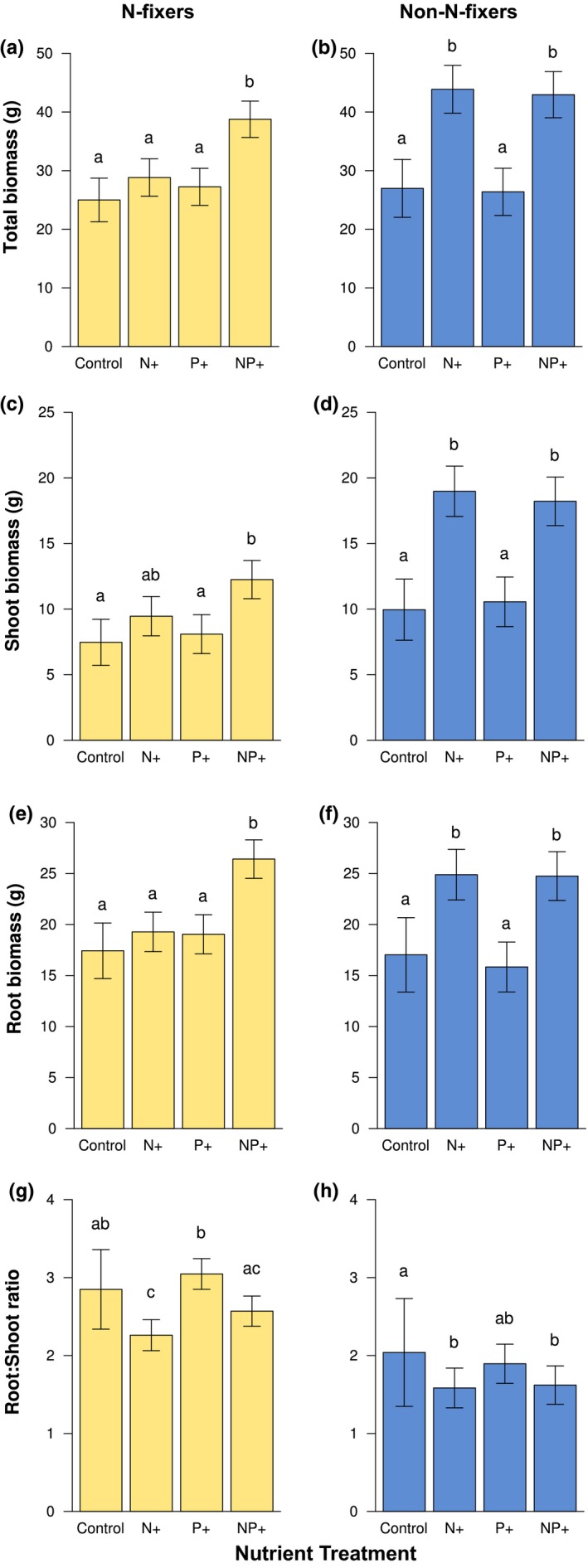
Response of N‐fixing and non‐N‐fixing species to N and P fertilization with respect to total biomass (a, b), aboveground biomass (c, d), belowground biomass accumulation (e, f), and root–shoot (R:S) ratios (g, h). Letters indicate significant differences within functional groups. Error bars represent 1 *SE*

N‐fixers and non‐N‐fixers differed in their response to nutrient addition (significant nutrient treatment x functional group interaction for total biomass: *F *=* *5.6984*, df *=* *3*, p *<* *0.001; shoot biomass: *F *=* *6.1188*, df *=* *3*, p *<* *0.001; root biomass: *F *=* *4.7438*, df *=* *3*, p *=* *0.003). N‐fixers in this system appeared to be co‐limited by N and P, showing significant increases in total (55%; *p *<* *0.001; Figure 1a), shoot (64%; *p *=* *0.001; Figure 1c), and root biomass (51%; *p *<* *0.001; Figure 1e) only when supplied with both N and P. Non‐N‐fixers, on the other hand, appeared to be N limited, with increases in total (63%; *p *<* *0.001; Figure 1b), shoot (91%; *p *<* *0.001; Figure 1d), and root biomass (46%; *p *<* *0.001; Figure 1f) observed for the N addition treatment, which were virtually identical to the increases observed in the NP+ treatment (59%, 83% and 45%, for total, shoot, and root biomass, respectively). P addition had no effect on biomass responses.

Differences in the responses of N‐fixers and non‐N‐fixers to nutrient addition were strongest for shoot biomass. N‐fixers increased their above‐ground biomass by 64% from approximately 7.47 g in the control treatment to 12.25 g in the NP+ treatment (Figure 1c), while biomass in non‐N‐fixers increased by 83% from 9.96 g to 18.22 g (Figure 1d). Similarly, total plant biomass increased by 55% from 25 g in the control treatment to 38.77 g in the NP+ treatment for N‐fixers (Figure 1a), while for the same treatment combinations, total plant biomass increased by 59% from 26.98 g to 42.97 g in non‐N‐fixers (Figure 1b). Increases in root biomass with nutrient addition were of similar magnitude for both functional groups (Figure 1e,f).

On average, there was no difference between the two functional groups in R:S ratios (*p *=* *0.27; Figure 1g,h). However, N‐fixers tended to invest relatively more in below‐ground tissue on average (R:S = 2.8, *SE *=* *0.51) compared to non‐N‐fixers (R:S = 2.04, *SE *=* *0.69). For both N‐fixers and non‐N‐fixers, R:S ratios declined in response to nutrient addition (nutrient treatment x functional group interaction: NS). However, within functional groups, N‐fixer (Figure 1g) R:S ratios declined by 21% with N addition (*p *=* *0.003) and by 10% in the NP+ treatment (NS), while non‐N‐fixers (Figure 1h) demonstrated significant declines in both the N+ (−22%; *p *=* *0.004) and NP+ treatments (−21%; *p *=* *0.007).

### 3.2 Root carbohydrate storage

**Figure 2:**
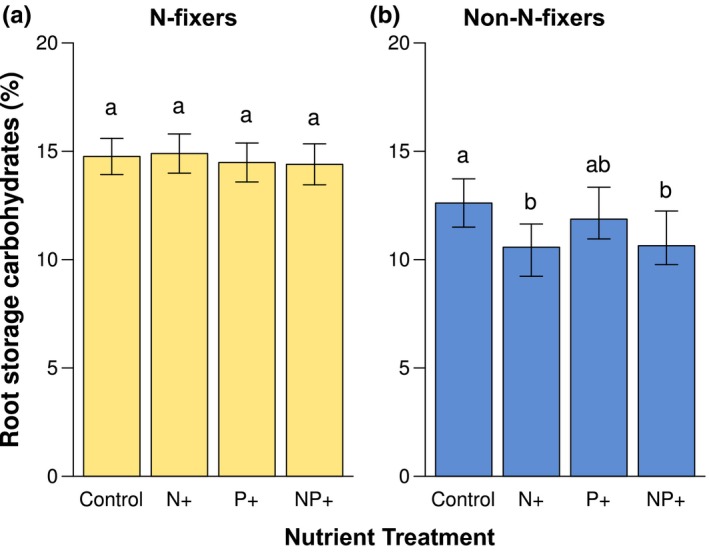
Modification in percent root storage carbohydrates in response to N and P addition for N‐fixing (a) and non‐N‐fixing species (b). Letters indicate significant differences within functional groups. Error bars represent 1 *SE*

N‐fixers had significantly greater concentrations of root carbohydrates compared to non‐N‐fixers (*p *=* *0.003, Figure 2). None of the nutrient treatments had any effect on root carbohydrate concentrations of N‐fixing species. In contrast, root carbohydrate concentrations in non‐N‐fixers declined significantly relative to controls following N addition, both when applied alone (N+) and in combination with P (i.e., NP+). P addition did not affect root carbohydrate concentrations (Figure 2b). For non‐N‐fixers, root storage carbohydrate contents declined from 12.6% in the controls to 10.6% in the N+ (*p *=* *0.01) and NP+ (*p *=* *0.01) treatments, a reduction of 16%.

### 3.3 Nodulation in N‐fixers

**Figure 3:**
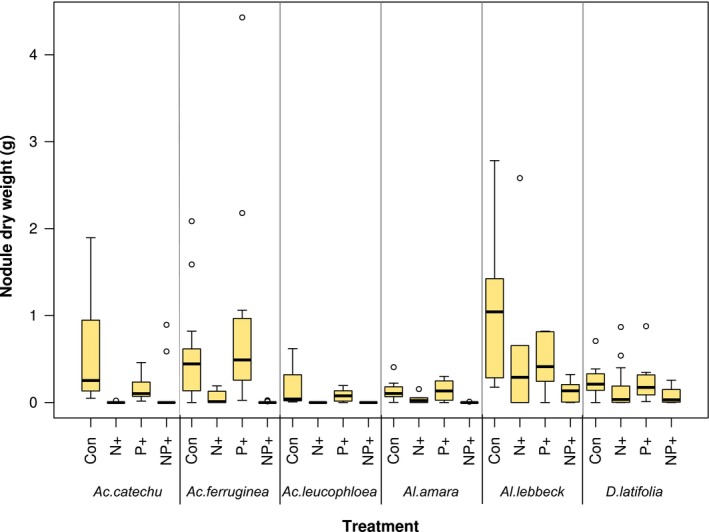
Changes in nodule dry weight in N‐fixing species in response to N and P addition

All N‐fixing species nodulated in the control treatment. Fertilization had a very strong negative effect on total nodule mass (species means) in N‐fixers (*F *=* *4.6622, *df *=* *3, *p *=* *0.02; Figure 3). Combined N and P addition resulted in the largest declines (−88%; *p *=* *0.004) in nodulation, followed by N addition (−67%; *p *=* *0.02). P addition resulted in marginal, but non‐significant reductions nodule weight.

## 4 DISCUSSION

Our results indicate that differences in the nature of nutrient limitation between N‐fixing and non‐N‐fixing tropical dry forest and savanna tree seedlings can lead to contrasting responses in the two functional groups to atmospheric nutrient deposition. Although seedlings of both functional groups increased biomass when fertilized, N‐fixers increased biomass only when simultaneously supplied with both N and P, while non‐N‐fixers responded only to N addition. Further, the magnitude of biomass increase with fertilization was greater for non‐N‐fixers when compared to N‐fixers. Baseline allocation in root biomass and root storage carbohydrates was greater in N‐fixers in our study, and although both functional groups tended to reduce relative investment in root biomass with nutrient addition, only non‐N‐fixers showed concurrent reductions in root storage carbohydrate content.

N‐fixers and non‐N‐fixers in our study differed in the nature of their nutrient limitation, with growth of non‐N‐fixing species limited by N availability, while N‐fixers were co‐limited by N and P in accordance with their N and P demanding lifestyle (Pearson & Vitousek, 2002; Vitousek et al., 2002, 2013). Previous studies that have investigated the nature of nutrient limitation of savanna vegetation have largely tended to focus on the herbaceous component of savannas (Barger, D'Antonio, Ghneim, Brink, & Cuevas, 2002; Bustamante et al., 2012; Cech, Kuster, Edwards, & Venterink, 2008; Copeland, Bruna, Silva, Mack, & Vasconcelos, 2012; Craine, Morrow, & Stock, 2008; Ludwig, de Kroon, Prins, & Berendse, 2001; O'Halloran et al., 2010; Ries & Shugart, 2008). Studies that have considered savanna trees have typically evaluated woody vegetation responses to the addition of a single nutrient (Kraaij & Ward, 2006; Wang, Katjiua, D'Odorico, & Okin, 2012), or the combined addition of N and P (Barbosa et al., 2014; van Der Waal et al., 2009; Khurana & Singh, 2004; Vadigi & Ward, 2013), thereby precluding identification of the specific nutrient(s) limiting growth (but see Wang et al., 2012; Holdo, 2013). Results from these earlier studies suggest contrasting patterns of nutrient limitation of herbaceous vegetation across the diverse savannas of the world, ranging from nutrients not being limiting (O'Halloran et al., 2010), to grass growth being N‐limited (Cech et al., 2008; Wang et al., 2012), P‐limited (Ludwig et al., 2001), or co‐limited by N and P (Cech et al., 2008; Craine et al., 2008). Woody plant responses, where evaluated, have also been similarly varied, with studies reporting no effects of N addition on growth of a non‐N‐fixing species (*Colophospermum mopane*; van Der Waal et al., 2009), P‐limitations to growth of an N‐fixing species (*Acacia erioloba*; Wang et al., 2012), and possible N and P co‐limitation of growth of a non‐N‐fixing species (*Combretum hereroense*; Holdo, 2013). There is also evidence to suggest that different life‐forms within a given savanna can be limited by different nutrients. For example, in a Namibian savanna, grass growth was reported to be N‐limited while trees displayed P‐limitation (Wang et al., 2012). While there have been surprisingly few studies that have investigated whether N‐fixing and non‐N‐fixing savanna trees differ in the nature of their nutrient limitation, results from this study and inferences based on differing N:P ratios of savanna grasses, N‐fixing, and non‐N‐fixing trees (Pellegrini, 2016; Ratnam, Sankaran, Hanan, Grant, & Zambatis, 2008) suggest that differential nutrient limitation of different plant functional groups in savannas may potentially be a common phenomenon. Ultimately, our results suggest that the effects of nutrient deposition on vegetation dynamics of mixed tree‐grass ecosystems is likely to be contingent on the relative rates of N and P being deposited in sites, with responses varying both spatially and between plant functional types within a site.

Non‐N‐fixers in our study were more responsive to the alleviation of nutrient limitation and showed greater relative increases in biomass with fertilization compared to N‐fixers. As expected, N‐fixers reduced investment in root nodules with increasing N availability (N+ and NP+ treatments; Gutschick, 1981; Sterner & Elser, 2002; Vitousek et al., 2002). Although resources that previously supported nodulation could have been diverted to further enhance growth (Boakye, Lawson, Owusu‐Bennoah, & Danso, 2015; Davidson & Robson, 1986), this was not reflected in the magnitude of biomass increase in N‐fixers observed in our study, which was less pronounced than that observed in non‐N‐fixers. These results are consistent with broader scale patterns reported in a meta‐analysis by Xia and Wan (2008), where biomass increases in response to fertilization in non‐N‐fixers were twice as large as those of N‐fixers across a range of plant life‐forms and ecosystem types. Differences in responses to fertilization between functional groups were particularly pronounced for shoot biomass, with non‐N‐fixers nearly doubling aboveground biomass (83% increase) with combined N and P addition, as opposed to a 64% increase in N‐fixers. Barbosa et al. (2014) also report similar results, where NPK fertilization resulted in increases in seedling stem length only among non‐N‐fixing South African savanna tree species. Greater relative investment in aboveground growth can be an effective strategy for juveniles to avoid light competition and quickly escape the zone of grass fuelled fires above which fire‐induced tree mortality is low (i.e., the fire trap), when fires are infrequent. However, it potentially comes at the cost of being able to survive, resprout, and persist within the fire trap when fires are frequent. Although both plant functional types in our study increased total investment in both above‐ and belowground tissues following fertilization, non‐N‐fixers reduced relative investment in belowground tissues (R:S ratios) to a greater extent than N‐fixers. Further, non‐N‐fixers reduced root carbohydrate stocks following fertilization, while N‐fixers adopted a more “conservative” strategy and continued to maintain their root carbohydrate reserves. Greater investment in belowground tissues (R:S ratios), and root carbohydrate stocks in particular, has been linked to faster rates of postfire recovery (Bell, 2001; Bond et al., 2003; Clarke & Knox, 2009; Clarke et al., 2013; Hoffmann et al., 2000; Lamont & Wiens, 2003; Vesk & Westoby, 2004; Wigley et al., 2009) suggesting that N‐fixing species are likely to be less prone to fire‐mediated mortality following fertilization. However, the extent to which enhanced nutrient availability can drive long‐term shifts in woody plant communities is likely to vary spatially, contingent on both local fire regimes (Aranibar et al., 2003; Frost & Robertson, 1985) and the relative availability of nutrients such as N and P in sites (Allen, 1964; Aranibar et al., 2003; Pivello & Coutinho, 1992; Pivello et al., 2010; Raison, 1979; Rundel & Parsons, 1984; Schafer & Mack, 2010; Singh, Raghubanshi & Singh, 1991).

Differences observed between N‐fixers and non‐N‐fixers in our study in terms of investment in root carbohydrate reserves following fertilization can potentially be attributed to differences in the type of reserves that the two functional groups invest in, that is, true and accumulated reserves (Chapin, Schulze, & Mooney, 1990). True reserves represent baseline plant investment in carbohydrate reserves, whose formation trades‐off with allocation of resources to plant growth, maintenance, and reproduction, and are not influenced by changes in resource availability (Clarke & Knox, 2009; Knox & Clarke, 2005). Accumulated reserves, on the other hand, are formed in addition to true reserves when the acquisition of non‐limiting resources exceeds demands for growth (Chapin et al., 1990). Plants increase their investment in accumulated reserves when faced with nutrient limitation for growth, but when an adequate supply of other resources is available for photosynthesis to continue. When supplied with limiting nutrients, carbohydrates previously contributing towards the formation of accumulated reserves are utilized to enhance growth. The lack of changes in root carbohydrate content with fertilization suggests that root carbohydrate reserves in N‐fixers may be predominantly made up of true reserves. In contrast, the reductions in percent root storage carbohydrates in non‐N‐fixers with N addition implies the formation of accumulated reserves in addition to true reserves, which may be associated with the larger increases in biomass production observed in non‐N‐fixers when fertilized.

Here, we investigated the responses of N‐fixing and non‐N‐fixing savanna and dry forest woody seedlings to atmospheric nutrient deposition—a pervasive, global change driver, focusing on patterns of seedling growth and biomass allocation when grown alone. While we recognize that ultimate responses are likely to be influenced by factors such as grass competition, feedback of fire on nutrient availability, as well as the interaction between plant nutrient status and herbivory, we nevertheless believe that our results provide a basis for understanding savanna responses to nutrient deposition. Our results suggest that nutrient deposition has the potential to induce longer‐term compositional shifts in savanna tree communities by differentially affecting the growth, postfire survival, and resprouting ability of N‐fixing and non‐N‐fixing species. Further, responses are likely to differ between sites contingent on multiple factors including underlying edaphic characteristics, fire regimes, and the relative amounts of N and P being deposited in sites.

## CONFLICT OF INTEREST

None declared.

## AUTHOR CONTRIBUTIONS

VV and MS designed the study. VV conducted the experiment, data collection, and analysis with inputs from MS. AMC calibrated and conducted the root storage carbohydrate assay with inputs from VV. VV wrote the manuscript with inputs from AMC and MS.

## DATA ACCESSIBILITY

Data available from the Dryad Digital Repository: https://doi.org/10.5061/dryad.09h8p8d.

## References

[ece34289-bib-0001] Allen, S. E. (1964). Chemical aspects of heather burning. Journal of Applied Ecology, 1, 347–367. 10.2307/2401318

[ece34289-bib-0002] Aranibar, J. N. , Macko, S. A. , Anderson, I. C. , Potgieter, A. L. F. , Sowry, R. , & Shugart, H. H. (2003). Nutrient cycling responses to fire frequency in the Kruger National Park (South Africa) as indicated by Stable Isotope analysis. Isotopes in Environmental and Health Studies, 39, 141–158. 10.1080/1025601031000096736 12872806

[ece34289-bib-0003] Barbosa, E. R. M. , van Langevelde, F. , Tomlinson, K. W. , Carvalheiro, L. G. , Kirkman, K. , de Bie, S. , & Prins, H. H. T. (2014). Tree species from different functional groups respond differently to environmental changes during establishment. Oecologia, 174, 1345–1357. 10.1007/s00442-013-2853-y 24337711

[ece34289-bib-0004] Barger, N. N. , D'Antonio, C. M. , Ghneim, T. , Brink, K. , & Cuevas, E. (2002). Nutrient limitation to primary productivity in a secondary savanna in Venezuela. Biotropica, 34, 493–501. 10.1111/j.1744-7429.2002.tb00569.x

[ece34289-bib-0005] Barron, A. R. , Purves, D. W. , & Hedin, L. O. (2010). Facultative nitrogen fixation by canopy legumes in a lowland tropical forest. Oecologia, 165, 511–520. 21110206 10.1007/s00442-010-1838-3

[ece34289-bib-0006] Bates, D. , Maechler, M. , Bolker, B. M. , & Walker, S. (2014). lme4: Linear mixed‐effects models using Eigen and S4. Journal of Statistical software, ArXiv e‐print http://arxiv.org/abs/1406.5823.

[ece34289-bib-0007] Bell, D. T. (2001). Ecological response syndromes in the flora of southwestern Western Australia: Fire resprouters versus reseeders. The Botanical Review, 67, 417–440. 10.1007/BF02857891

[ece34289-bib-0008] Bell, T. L. , & Ojeda, F. (1999). Underground starch storage in *Erica* species of the Cape Floristic Region – differences between seeders and resprouters. New Phytologist, 144, 143–152. 10.1046/j.1469-8137.1999.00489.x

[ece34289-bib-0009] Bennett, E. M. , Carpenter, S. R. , & Caraco, N. F. (2001). Human impact on erodable phosphorus and eutrophication: A global perspective increasing accumulation of phosphorus in soil threatens rivers, lakes, and coastal oceans with eutrophication. BioScience, 51, 227–234. 10.1641/0006-3568(2001)051[0227:HIOEPA]2.0.CO;2

[ece34289-bib-0010] Boakye, E. Y. , Lawson, I. Y. D. , Owusu‐Bennoah, E. , & Danso, S. K. A. (2015). Growth and nodulation response of six indigenous trees and two shrubby legumes to phosphorus and nitrogen fertilizers in two soils of Ghana. Journal of Tropical Agriculture, 53, 21–34.

[ece34289-bib-0011] Bond, W. J. (2008). What limits trees in C_4_ grasslands and savannas? Annual Review of Ecology, Evolution, and Systematics, 39, 641–659. 10.1146/annurev.ecolsys.39.110707.173411

[ece34289-bib-0012] Bond, W. J. , Midgley, G. F. , Woodward, F. I. , Hoffman, M. T. , & Cowling, R. M. (2003). What controls South African vegetation — climate or fire? South African Journal of Botany, 69, 79–91. 10.1016/S0254-6299(15)30362-8

[ece34289-bib-0013] Bond, W. J. , & van Wilgen, B. W. (1996). Fire and plants. Netherlands: Springer 10.1007/978-94-009-1499-5

[ece34289-bib-0014] Bustamante, M. M. C. , Brito, D. Q. de. , Kozovits, A. R. , Luedemann, G. , Mello, T. R. B. de. , Pinto, A. de. S. , … Takahashi, F. S. C. (2012). Effects of nutrient additions on plant biomass and diversity of the herbaceous‐subshrub layer of a Brazilian savanna (Cerrado). Plant Ecology, 213, 795–808. 10.1007/s11258-012-0042-4

[ece34289-bib-0015] Buysse, J. , & Merckx, R. (1993). An improved colorimetric method to quantify sugar content of plant tissue. Journal of Experimental Botany, 44, 1627–1629. 10.1093/jxb/44.10.1627

[ece34289-bib-0016] Cech, P. G. , Kuster, T. , Edwards, P. J. , & Venterink, H. O. (2008). Effects of herbivory, fire and N_2_‐fixation on nutrient limitation in a humid African savanna. Ecosystems, 11, 991–1004. 10.1007/s10021-008-9175-7

[ece34289-bib-0017] Chapin, F. S. , Schulze, E.‐D. , & Mooney, H. A. (1990). The ecology and economics of storage in plants. Annual Review of Ecology and Systematics, 21, 423–447. 10.1146/annurev.es.21.110190.002231

[ece34289-bib-0018] Clarke, P. J. , & Knox, K. J. E. (2009). Trade‐offs in resource allocation that favour resprouting affect the competitive ability of woody seedlings in grassy communities. Journal of Ecology, 97, 1374–1382. 10.1111/j.1365-2745.2009.01556.x

[ece34289-bib-0019] Clarke, P. J. , Lawes, M. J. , Midgley, J. J. , Lamont, B. B. , Ojeda, F. , Burrows, G. E. , … Knox, K. J. E. (2013). Resprouting as a key functional trait: How buds, protection and resources drive persistence after fire. New Phytologist, 197, 19–35. 10.1111/nph.12001 23110592

[ece34289-bib-0020] Copeland, S. M. , Bruna, E. M. , Silva, L. V. B. , Mack, M. C. , & Vasconcelos, H. L. (2012). Short‐term effects of elevated precipitation and nitrogen on soil fertility and plant growth in a neotropical savanna. Ecosphere, 3, 1–20.

[ece34289-bib-0021] Craine, J. M. , Morrow, C. , & Stock, W. D. (2008). Nutrient concentration ratios and co‐limitation in South African grasslands. New Phytologist, 179, 829–836. 10.1111/j.1469-8137.2008.02513.x 18537887

[ece34289-bib-0022] Cramer, M. D. , Chimphango, S. B. M. , Van Cauter, A. , Waldram, M. S. , & Bond, W. J. (2007). Grass competition induces N_2_ fixation in some species of African *Acacia* . Journal of Ecology, 95, 1123–1133. 10.1111/j.1365-2745.2007.01285.x

[ece34289-bib-0023] Cramer, M. D. , Van Cauter, A. , & Bond, W. J. (2010). Growth of N_2_‐fixing African savanna *Acacia* species is constrained by below‐ground competition with grass. Journal of Ecology, 98, 156–167. 10.1111/j.1365-2745.2009.01594.x

[ece34289-bib-0024] Davidson, I. A. , & Robson, M. J. (1986). Effect of contrasting patterns of nitrate application on the nitrate uptake, N_2_‐fixation, nodulation and growth of white clover. Annals of Botany, 57, 331–338. 10.1093/oxfordjournals.aob.a087114

[ece34289-bib-0025] Ekblom, A. , & Gillson, L. (2010). Hierarchy and scale: Testing the long term role of water, grazing and nitrogen in the savanna landscape of Limpopo National Park (Mozambique). Landscape Ecology, 25, 1529–1546. 10.1007/s10980-010-9522-x

[ece34289-bib-0026] February, E. C. , & Higgins, S. I. (2010). The distribution of tree and grass roots in savannas in relation to soil nitrogen and water. South African Journal of Botany, 76, 517–523. 10.1016/j.sajb.2010.04.001

[ece34289-bib-0027] February, E. C. , Higgins, S. I. , Newton, R. , & West, A. G. (2007). Tree distribution on a steep environmental gradient in an arid savanna. Journal of Biogeography, 34, 270–278. 10.1111/j.1365-2699.2006.01583.x

[ece34289-bib-0028] Frost, P. G. H. , Medina, E. , Menaut, J. , Solbrig, O. , Swift, M. , & Walker, B. H. (1986). Response of savannas to stress and disturbance. Biology International Special Issue 10. Paris, France: IUBS.

[ece34289-bib-0029] Frost, P. G. H. , & Robertson, F. (1985). Fire the ecological effects of fire in savannas In Walker T. S. & Walker B. H. (Eds.), Determinants of tropical savannas (pp. 93–140). Oxford, UK: IUBS.

[ece34289-bib-0030] Galloway, J. N. , Dentener, F. J. , Capone, D. G. , Boyer, E. W. , Howarth, R. W. , Seitzinger, S. P. , … Vöosmarty, C. J. (2004). Nitrogen cycles: Past, present, and future. Biogeochemistry, 70, 153–226. 10.1007/s10533-004-0370-0

[ece34289-bib-0031] Galloway, J. N. , Townsend, A. R. , Erisman, J. W. , Bekunda, M. , Cai, Z. , Freney, J. R. , … Sutton, M. A. (2008). Transformation of the nitrogen cycle: Recent trends, questions, and potential solutions. Science, 320, 889–892. 10.1126/science.1136674 18487183

[ece34289-bib-0032] Graham, P. H. , & Vance, C. P. (2003). Legumes: Importance and constraints to greater use. Plant Physiology, 131, 872–877. 10.1104/pp.017004 12644639 PMC1540286

[ece34289-bib-0033] Gutschick, V. P. (1981). Evolved strategies in nitrogen acquisition by plants. The American Naturalist, 118, 607–637. 10.1086/283858

[ece34289-bib-0034] Hanan, N. P. , Sea, W. B. , Dangelmayr, G. , & Govender, N. (2008). Do fires in savannas consume woody biomass? A comment on approaches to modeling savanna dynamics. The American Naturalist, 171, 851–856. 10.1086/587527 18462133

[ece34289-bib-0035] Hermans, C. , Hammond, J. P. , White, P. J. , & Verbruggen, N. (2006). How do plants respond to nutrient shortage by biomass allocation? Trends in Plant Science, 11, 610–617. 10.1016/j.tplants.2006.10.007 17092760

[ece34289-bib-0036] Higgins, S. I. , Bond, W. J. , & Trollope, W. S. W. (2000). Fire, resprouting and variability: A recipe for grass–tree coexistence in savanna. Journal of Ecology, 88, 213–229. 10.1046/j.1365-2745.2000.00435.x

[ece34289-bib-0037] Hoffmann, W. A. , Bazzaz, F. A. , Chatterton, N. J. , Harrison, P. A. , & Jackson, R. B. (2000). Elevated CO_2_ enhances resprouting of a tropical savanna tree. Oecologia, 123, 312–317. 10.1007/s004420051017 28308585

[ece34289-bib-0038] Holdo, R. M. (2013). Effects of fire history and N and P fertilization on seedling biomass, Specific Leaf Area, and root:Shoot ratios in a South African savannah. South African Journal of Botany, 86, 5–8. 10.1016/j.sajb.2013.01.005

[ece34289-bib-0039] Khurana, E. , & Singh, J. S. (2004). Impact of elevated nitrogen inputs on seedling growth of five dry tropical tree species as affected by life‐history traits. Canadian Journal of Botany, 82, 158–167. 10.1139/b03-132

[ece34289-bib-0040] Knox, K. J. E. , & Clarke, P. J. (2005). Nutrient availability induces contrasting allocation and starch formation in resprouting and obligate seeding shrubs. Functional Ecology, 19, 690–698. 10.1111/j.1365-2435.2005.01006.x

[ece34289-bib-0041] Kodandapani, N. , Cochrane, M. A. , & Sukumar, R. (2008). A comparative analysis of spatial, temporal, and ecological characteristics of forest fires in seasonally dry tropical ecosystems in the Western Ghats, India. Forest Ecology and Management, 256, 607–617. 10.1016/j.foreco.2008.05.006

[ece34289-bib-0042] Kraaij, T. , & Ward, D. (2006). Effects of rain, nitrogen, fire and grazing on tree recruitment and early survival in bush‐encroached savanna, South Africa. Plant Ecology, 186, 235–246. 10.1007/s11258-006-9125-4

[ece34289-bib-0043] Kumar, R. , & Shahabuddin, G. (2005). Effects of biomass extraction on vegetation structure, diversity and composition of forests in Sariska Tiger Reserve, India. Environmental Conservation, 32, 248–259. 10.1017/S0376892905002316

[ece34289-bib-0044] Kuznetova, A. , Brockhoff, P. , & Christensen, R. J. (2014). lmerTest: Tests for random and fixed effects for linear mixed effect models (lmer objects of lme4 package). R package version 2.0‐11.

[ece34289-bib-0045] Lamont, B. B. , & Wiens, D. (2003). Are seed set and speciation rates always low among species that resprout after fire, and why? Evolutionary Ecology, 17, 277–292. 10.1023/A:1025535223021

[ece34289-bib-0046] Lehmann, C. E. R. , Anderson, T. M. , Sankaran, M. , Higgins, S. I. , Archibald, S. , Hoffmann, W. A. , … Bond, W. J. (2014). Savanna vegetation‐fire‐climate relationships differ among continents. Science, 343, 548–552. 10.1126/science.1247355 24482480

[ece34289-bib-0047] Ludwig, F. , de Kroon, H. , Prins, H. H. T. , & Berendse, F. (2001). Effects of nutrients and shade on tree‐grass interactions in an East African savanna. Journal of Vegetation Science, 12, 579–588. 10.2307/3237009

[ece34289-bib-0048] O'Halloran, L. R. , Shugart, H. H. , Wang, L. , Caylor, K. K. , Ringrose, S. , & Kgope, B. (2010). Nutrient limitations on aboveground grass production in four savanna types along the Kalahari Transect. Journal of Arid Environments, 74, 284–290. 10.1016/j.jaridenv.2009.08.012

[ece34289-bib-0049] Pate, J. S. , Froend, R. H. , Bowen, B. J. , Hansen, A. , & Kuo, J. (1990). seedling growth and storage characteristics of seeder and resprouter species of Mediterranean‐type ecosystems of S.W. Australia. Annals of Botany, 65, 585–601. 10.1093/oxfordjournals.aob.a087976

[ece34289-bib-0050] Pearson, H. L. , & Vitousek, P. M. (2002). Soil phosphorus fractions and symbiotic nitrogen fixation across a substrate‐age gradient in Hawaii. Ecosystems, 5, 587–596. 10.1007/s10021-002-0172-y

[ece34289-bib-0051] Pellegrini, A. F. A. (2016). Nutrient limitation in tropical savannas across multiple scales and mechanisms. Ecology, 97, 313–324. 10.1890/15-0869.1 27145607

[ece34289-bib-0052] Phoenix, G. K. , Hicks, W. K. , Cinderby, S. , Kuylenstierna, J. C. I. , Stock, W. D. , Dentener, F. J. , … Ineson, P. (2006). Atmospheric nitrogen deposition in world biodiversity hotspots: The need for a greater global perspective in assessing N deposition impacts. Global Change Biology, 12, 470–476. 10.1111/j.1365-2486.2006.01104.x

[ece34289-bib-0053] Pivello, V. R. , & Coutinho, L. M. (1992). Transfer of macro‐nutrients to the atmosphere during experimental burnings in an open cerrado (Brazilian savanna). Journal of Tropical Ecology, 8, 487–497. 10.1017/S0266467400006829

[ece34289-bib-0054] Pivello, Vânia. Regina. , Oliveras, I. , Miranda, H. S. , Haridasan, M. , Sato, M. N. , & Meirelles, S. T. (2010). Effect of fires on soil nutrient availability in an open savanna in Central Brazil. Plant and Soil, 337, 111–123. 10.1007/s11104-010-0508-x

[ece34289-bib-0055] Powers, J. S. , & Tiffin, P. (2010). Plant functional type classifications in tropical dry forests in Costa Rica: Leaf habit versus taxonomic approaches. Functional Ecology, 24, 927–936. 10.1111/j.1365-2435.2010.01701.x

[ece34289-bib-0056] Puyravaud, J.‐P. , Pascal, J.‐P. , & Dufour, C. (1994). Ecotone structure as an indicator of changing forest‐savanna boundaries (Linganamakki region, southern India). Journal of Biogeography, 21, 581–593. 10.2307/2846033

[ece34289-bib-0057] R Core Team . (2014). R: A language and environment for statistical computing. Vienna, Austria: R Foundation for Statistical Computing Retrieved from http://www.R-project.org/

[ece34289-bib-0058] Raison, R. J. (1979). Modification of the soil environment by vegetation fires, with particular reference to nitrogen transformations: A review. Plant and Soil, 51, 73–108. 10.1007/BF02205929

[ece34289-bib-0059] Ratnam, J. , Sankaran, M. , Hanan, N. P. , Grant, R. C. , & Zambatis, N. (2008). Nutrient resorption patterns of plant functional groups in a tropical savanna: Variation and functional significance. Oecologia, 157, 141–151. 10.1007/s00442-008-1047-5 18488252

[ece34289-bib-0060] Ries, L. P. , & Shugart, H. H. (2008). Nutrient limitations on understory grass productivity and carbon assimilation in an African woodland savanna. Journal of Arid Environments, 72, 1423–1430. 10.1016/j.jaridenv.2008.02.013

[ece34289-bib-0061] Rundel, P. W. , & Parsons, D. J. (1984). Post‐fire uptake of nutrients by diverse ephemeral herbs in chamise chaparral. Oecologia, 61, 285–288. 10.1007/BF00396774 28309425

[ece34289-bib-0062] Ryle, G. J. A. , Arnott, R. A. , & Powell, C. E. (1981). Distribution of dry weight between root and shoot in white clover dependent on N_2_ fixation or utilizing abundant nitrate nitrogen. Plant and Soil, 60, 29–39. 10.1007/BF02377110

[ece34289-bib-0063] Sagar, R. , & Singh, J. S. (2004). Local plant species depletion in a tropical dry deciduous forest of northern India. Environmental Conservation, 31, 55–62. 10.1017/S0376892904001031

[ece34289-bib-0064] Sala, O. E. , Chapin, F. S. III , Armesto, J. J. , Berlow, E. , Bloomfield, J. , Dirzo, R. , … Wall, D. H. (2000). Global biodiversity scenarios for the year 2100. Science, 287, 1770–1774. 10.1126/science.287.5459.1770 10710299

[ece34289-bib-0065] Sanginga, N. , Mulongoy, K. , & Ayanaba, A. (1988). Nodulation and growth of *Leucaena leucocephala* (Lam.) de Wit as affected by inoculation and N fertilizer. Plant and Soil, 112, 129–135. 10.1007/BF02181762

[ece34289-bib-0066] Sankaran, M. , Hanan, N. P. , Scholes, R. J. , Ratnam, J. , Augustine, D. J. , Cade, B. S. , … Zambatis, N. (2005). Determinants of woody cover in African savannas. Nature, 438, 846–849. 10.1038/nature04070 16341012

[ece34289-bib-0067] Sankaran, M. , Ratnam, J. , & Hanan, N. P. (2004). Tree–grass coexistence in savannas revisited – insights from an examination of assumptions and mechanisms invoked in existing models. Ecology Letters, 7, 480–490. 10.1111/j.1461-0248.2004.00596.x

[ece34289-bib-0068] Sankaran, M. , Ratnam, J. , & Hanan, N. P. (2008). Woody cover in African savannas: The role of resources, fire and herbivory. Global Ecology and Biogeography, 17, 236–245. 10.1111/j.1466-8238.2007.00360.x

[ece34289-bib-0069] Schafer, J. L. , & Mack, M. C. (2010). Short‐term effects of fire on soil and plant nutrients in palmetto flatwoods. Plant and Soil, 334, 433–447. 10.1007/s11104-010-0394-2

[ece34289-bib-0070] Scholes, R. J. , & Archer, S. R. (1997). Tree‐grass interactions in savannas. Annual Review of Ecology and Systematics, 28, 517–544. 10.1146/annurev.ecolsys.28.1.517

[ece34289-bib-0071] Schutz, A. E. N. , Bond, W. J. , & Cramer, M. D. (2009). Juggling carbon: Allocation patterns of a dominant tree in a fire‐prone savanna. Oecologia, 160, 235–246. 10.1007/s00442-009-1293-1 19214583

[ece34289-bib-0072] Schwilk, D. W. , & Ackerly, D. D. (2005). Is there a cost to resprouting? Seedling growth rate and drought tolerance in sprouting and nonsprouting *Ceanothus* (Rhamnaceae). American Journal of Botany, 92, 404–410. 10.3732/ajb.92.3.404 21652416

[ece34289-bib-0073] Singh, R. S. , Raghubanshi, A. S. , & Singh, J. S. , (1991). Nitrogen‐mineralization in dry tropical savanna: Effects of burning and grazing. Soil Biology and Biochemistry, 23, 269–273. 10.1016/0038-0717(91)90063-P

[ece34289-bib-0074] Sprent, J. I. (1999). Nitrogen fixation and growth of non‐crop legume species in diverse environments. Perspectives in Plant Ecology, Evolution and Systematics, 2, 149–162. 10.1078/1433-8319-00068

[ece34289-bib-0075] Sterner, R. W. , & Elser, J. J. (2002). Ecological stoichiometry: The biology of elements from molecules to the biosphere. New Jersey, USA: Princeton University Press.

[ece34289-bib-0076] Tilman, D. (1988). Plant strategies and the dynamics and structure of plant communities. New Jersey, USA: Princeton University Press.

[ece34289-bib-0077] Tomlinson, K. W. , van Langevelde, F. , Ward, D. , Bongers, F. , da Silva, D. A. , Alves, D. , … Sterck, F. J. (2013). Deciduous and evergreen trees differ in juvenile biomass allometries because of differences in allocation to root storage. Annals of Botany, 112(3), 575–587. 10.1093/aob/mct132 23877001 PMC3718220

[ece34289-bib-0078] Vadigi, S. , & Ward, D. (2013). Shade, nutrients, and grass competition are important for tree sapling establishment in a humid savanna. Ecosphere, 4, art142 10.1890/ES13-00239.1

[ece34289-bib-0079] Verdaguer, D. , & Ojeda, F. (2002). Root starch storage and allocation patterns in seeder and resprouter seedlings of two Cape Erica (*Ericaceae*) species. American Journal of Botany, 89, 1189–1196. 10.3732/ajb.89.8.1189 21665719

[ece34289-bib-0080] van Der Waal, C. , De Kroon, H. , De Boer, W. F. , Heitkönig, I. M. A. , Skidmore, A. K. , De Knegt, H. J. , … Prins, H. H. T. (2009). Water and nutrients alter herbaceous competitive effects on tree seedlings in a semi‐arid savanna. Journal of Ecology, 97, 430–439. 10.1111/j.1365-2745.2009.01498.x

[ece34289-bib-0081] van der Waal, C. , Kool, A. , Meijer, S. S. , Kohi, E. , Heitkönig, I. M. A. , de Boer, W. F. , … de Kroon, H. (2011). Large herbivores may alter vegetation structure of semi‐arid savannas through soil nutrient mediation. Oecologia, 165, 1095–1107. 10.1007/s00442-010-1899-3 21225433 PMC3057003

[ece34289-bib-0200] Vance, C. P. , Graham, P. H. , Allan, D. L. , Pedrosa, F. O. , Hungria, M. , Yates, G. , & Newton, W. E. (2000). Biological nitrogen fixation: Phosphorus ‐ A critical future need? In Nitrogen fixation: From molecules to crop productivity (pp. 509–514). Netherlands: Springer.

[ece34289-bib-0082] Vesk, P. A. , & Westoby, M. (2004). Funding the bud bank: A review of the costs of buds. Oikos, 106, 200–208. 10.1111/j.0030-1299.2004.13204.x

[ece34289-bib-0083] Vitousek, P. M. (1994). Beyond global warming: Ecology and global change. Ecology, 75, 1861–1876. 10.2307/1941591

[ece34289-bib-0084] Vitousek, P. M. , Aber, J. D. , Howarth, R. W. , Likens, G. E. , Matson, P. A. , Schindler, D. W. , … Tilman, D. G. (1997). Human alteration of the global nitrogen cycle: Sources and consequences. Ecological Applications, 7, 737–750.

[ece34289-bib-0085] Vitousek, P. M. , Cassman, K. , Cleveland, C. , Crews, T. , Field, C. B. , Grimm, N. B. , … Sprent, J. I. (2002). Towards an ecological understanding of biological nitrogen fixation. Biogeochemistry, 57–58, 1–45. 10.1023/A:1015798428743

[ece34289-bib-0086] Vitousek, P. M. , & Howarth, R. W. (1991). Nitrogen limitation on land and in the sea: How can it occur? Biogeochemistry, 13, 87–115.

[ece34289-bib-0087] Vitousek, P. M. , Menge, D. N. L. , Reed, S. C. , & Cleveland, C. C. (2013). Biological nitrogen fixation: Rates, patterns and ecological controls in terrestrial ecosystems. Philosophical Transactions of the Royal Society B: Biological Sciences, 368, 20130119 10.1098/rstb.2013.0119 PMC3682739 23713117

[ece34289-bib-0088] Vitousek, P. M. , Porder, S. , Houlton, B. Z. , & Chadwick, O. A. (2010). Terrestrial phosphorus limitation: Mechanisms, implications, and nitrogen–phosphorus interactions. Ecological Applications, 20, 5–15. 10.1890/08-0127.1 20349827

[ece34289-bib-0089] Wang, Y.‐L. , Almvik, M. , Clarke, N. , Eich‐Greatorex, S. , Øgaard, A. F. , Krogstad, T. , … Clarke, J. L. (2015). Contrasting responses of root morphology and root‐exuded organic acids to low phosphorus availability in three important food crops with divergent root traits. AoB Plants, 7 10.1093/aobpla/plv097 PMC4583607 26286222

[ece34289-bib-0090] Wang, L. , Katjiua, M. , D'Odorico, P. , & Okin, G. S. (2012). The interactive nutrient and water effects on vegetation biomass at two African savannah sites with different mean annual precipitation. African Journal of Ecology, 50, 446–454. 10.1111/j.1365-2028.2012.01339.x

[ece34289-bib-0091] Wigley, B. J. , Cramer, M. D. , & Bond, W. J. (2009). Sapling survival in a frequently burnt savanna: Mobilisation of carbon reserves in *Acacia karroo* . Plant Ecology, 203, 1–11. 10.1007/s11258-008-9495-x

[ece34289-bib-0092] Xia, J. , & Wan, S. (2008). Global response patterns of terrestrial plant species to nitrogen addition. New Phytologist, 179, 428–439. 10.1111/j.1469-8137.2008.02488.x 19086179

