# Supplementary material for: Effects of increased N and P availability on biomass allocation and root carbohydrate reserves differ between N‐fixing and non‐N‐fixing savanna tree seedlings
Source: Ecol Evol. 2018 Jul 30;8(16):8467–76. doi: 10.1002/ece3.4289 (PMC6144997; doi:10.1002/ece3.4289)
Supplement: Supplementary file 1 [file ECE3-8-8467-s001.pdf]

Supplementary table S1. Number of species-treatment replicates in the entire experiments (a) and for the root storage carbohydrate assay (b).

a)

	**Species**	**Treatment**			
		Control	N+	P+	NP+
N-fixers	*Acacia catechu*	13	12	13	12
	Acacia ferruginea	14	12	14	14
	Acacia leucophloea	5	5	5	5
	Albizia amara	7	6	8	7
	Albizia lebbeck	7	6	7	7
	Dalbergia latifolia	14	14	14	12
Non-N-fixers	*Lagerstroemia indica*	18	5	18	11
	Lagerstroemia speciosa	13	12	13	12
	Phyllanthus emblica	14	14	14	14
	Sapindus emarginatus	14	14	14	14
	Terminalia arjuna	12	13	11	11
	Terminalia bellirica	14	13	15	14
	Zizyphus jujuba	14	13	14	13

b)

	**Species**	**Treatment**			
		Control	N+	P+	NP+
N-fixers	*Acacia catechu*	6	6	7	6
	Acacia ferruginea	6	6	7	5
	Acacia leucophloea	5	4	4	2
	Albizia amara	5	6	6	5
	Albizia lebbeck	4	5	4	4
	Dalbergia latifolia	6	6	6	6
Non-N-fixers	*Lagerstroemia indica*	7	5	7	5
	Lagerstroemia speciosa	6	6	6	6
	Phyllanthus emblica	6	6	6	6
	Sapindus emarginatus	6	6	7	6
	Terminalia arjuna	6	6	6	6
	Terminalia bellirica	6	6	6	6
	Zizyphus jujuba	6	6	5	6
